# Ploidy influences wheat mesophyll cell geometry, packing and leaf function

**DOI:** 10.1002/pld3.314

**Published:** 2021-04-07

**Authors:** Matthew J. Wilson, Marc Fradera‐Soler, Richard Summers, Craig J. Sturrock, Andrew J. Fleming

**Affiliations:** ^1^ Department of Animal and Plant Sciences University of Sheffield Sheffield UK; ^2^ Department of Plant and Environmental Sciences University of Copenhagen Copenhagen Denmark; ^3^ RAGT Seeds Ltd Ickleton Cambridgeshire UK; ^4^ Hounsfield Facility Division of Agriculture and Environmental Sciences School of Biosciences University of Nottingham Sutton Bonington UK

**Keywords:** cell geometry, mesophyll, ploidy, water‐use efficiency, wheat (*Triticum aestivum*), X‐ray Computed Tomography

## Abstract

Leaf function is influenced by leaf structure, which is itself related not only to the spatial arrangement of constituent mesophyll cells, but also their size and shape. In this study, we used confocal microscopy to image leaves of *Triticum* genotypes varying in ploidy level to extract 3D information on individual mesophyll cell size and geometry. Combined with X‐ray Computed Tomography and gas exchange analysis, the effect of changes in wheat mesophyll cell geometry upon leaf structure and function were investigated. Mesophyll cell size and shape were found to have changed during the course of wheat evolution. An unexpected linear relationship between mesophyll cell surface area and volume was discovered, suggesting anisotropic scaling of mesophyll cell geometry with increasing ploidy. Altered mesophyll cell size and shape were demonstrated to be associated with changes in mesophyll tissue architecture. Under experimental growth conditions, CO_2_ assimilation did not vary with ploidy, but stomatal conductance was lower in hexaploid plants, conferring a greater instantaneous water‐use efficiency. We propose that as wheat mesophyll cells have become larger with increased ploidy, this has been accompanied by changes in cell geometry and packing which limit water loss while maintaining carbon assimilation.

## 1 INTRODUCTION

Understanding how leaves are constructed, and how this influences or limits the function of the leaf in terms of the primary function of photosynthesis, remains a central problem in plant biology (Earles, Buckley, et al., 2018; Ren et al., 2019; Terashima et al., 2011). For example, leaf mesophyll cells are core to the process of carbon assimilation and require both sufficient light and CO_2_ to perform this function, yet the supply of these raw materials depends to a large extent on structural properties at the whole leaf level, properties which are themselves influenced by the size and geometry of the constituent mesophyll cells and how they are arranged in space. With respect to gas exchange, the entry of carbon dioxide into the leaf's internal environment is controlled by stomata, which are also responsible for managing the concurrent loss of water vapor (Harrison et al., 2020; Lawson & Matthews, 2020). A complex pattern of airspaces created by the controlled separation of mesophyll cells then allows the flux of CO_2_ to these cells where diffusion across the cell wall, plasma membrane, and cytosol must occur before the entry of CO_2_ into chloroplasts, the site of carboxylation (Flexas et al., 2012; Lundgren & Fleming, 2020). However, the arrangement, size, shape, and separation of mesophyll cells influences not only CO_2_ flux, but also other parameters of photosynthesis (such as light capture) (Terashima et al., 2011; Tholen et al., 2012) and important elements linked to leaf structure and function, such as mechanical integrity and water flux (Buckley et al., 2015). Moreover, the process of development to generate these complex structures generally occurs in a changing environment where the “optimal” structure will involve numerous trade‐offs related to the life strategy of the plant and the specific environment in which it is growing (Brodribb et al., 2013; Flexas & Carriqui, 2020; Roddy et al., 2020).

Evolutionary approaches provide powerful tools to investigate this problem since there is often relatively large variation in individual cellular architectures centered around a consistent theme (i.e., the overall leaf structure and plant habit is conserved yet the constituent cells and development show variation upon this theme). In this respect, bread wheat (*T. aestivum*) provides an interesting experimental system. Not only does it provide a large range of extant modern and more ancient varieties, reflecting the evolution of modern hexaploid (6n) wheat from diploid (2n) and tetraploid (4n) species (El Baidouri et al., 2017), due to its importance as a staple crop there is the potential for findings on basic aspects of leaf form and function to be translated into agronomic use (Balfourier et al., 2019). Moreover, the rapid development of molecular genetic tools is opening the door to experimental analyses in wheat that until recently would not have been possible (Borrill et al., 2018; International Wheat Genome Sequencing Consortium, 2018; Uauy, 2017).

Wheat leaves display a similar basic cellular arrangement across each of the three ploidy levels. Unlike in many eudicot species, there is no differentiation into distinct palisade and spongy layers across the adaxial/abaxial axis. Instead, mesophyll cells are patterned along the proximal‐distal axis of the leaf, separated laterally across the width of the leaf by a series of major and minor veins (Jellings & Leech, 1984; Parker & Ford, 1982). The question of how wheat mesophyll size and shape has altered during the evolution/selection of modern wheat has been studied previously via a series of key papers revealing that as ploidy has increased, so has cell size (Jellings & Leech, 1984; Parker & Ford, 1982), a general effect of polyploidy often manifested in plant species. However, these studies were restricted by the imaging and data analysis processes available at the time. Essentially this involved separating the mesophyll cells from the leaf (thus losing information on how the cells were patterned in space), and required the deduction of 3D cell shape and volume from the analysis of 2D images (Dunstone & Evans, 1974; Jellings & Leech, 1984; Parker & Ford, 1982; Pyke et al., 1990). As well as being time‐consuming due to manual processing, these approaches do not provide full insight into 3D cell shape, and may lead to inaccuracies in estimates of cell/tissue parameters (Earles, Theroux‐Rancourt, et al., 2018; Harwood et al., 2020; Mathers et al., 2018; Théroux‐Rancourt et al., 2017) and thus less insight into the relationships between leaf form and function. In addition, modern 3D imaging methodologies allow for better inference of the complex multidimensional diffusion pathways associated with the exchange of carbon and water required for photosynthesis, enabling new insights into models of photosynthesis (Earles, Buckley, et al., 2018; Lehmeier et al., 2017). When linked with the more advanced technologies now available for the measurement of an array of parameters defining photosynthesis and water use, a deeper understanding of the potential link of mesophyll cell size, geometry, and leaf function is afforded.

In this paper, we describe a series of experiments in which we have investigated the evolution of wheat mesophyll cell size and shape by applying confocal and computed microtomography (microCT) imaging technologies with modern image segmentation and analytical tools to obtain quantitative data from 3D images (Barbier de Reuille et al., 2015; Pajor et al., 2013). Combining this with physiological analyses, we show that the evolution of the wheat mesophyll has been accompanied by complex changes in the relationship of surface area to the volume at the level of both the cell and tissue, with important implications for leaf physiology. In addition, we report on an apparent transition within tetraploid (4n) species with respect to water‐use efficiency following the switch to domestication.

## 2 MATERIALS AND METHODS

### 2.1 Plant materials and growth conditions

Seeds of a range of lines of *Triticum* spp.—listed in Table 1 (wild accessions obtained from IPK Gatersleben, Germany (https://www.ipk‐gatersleben.de) domesticated cultivars provided by RAGT Seeds were sown into module trays containing a mixture of 6:1 Levington M3 compost: Perlite and placed into a controlled environment growth chamber (Conviron PGR15; Conviron, Winnipeg). After germination, seedlings were transplanted to 1L pots containing the same compost mix, with the addition of five grams of solid slow‐release fertilizer (Osmocote Exact 5‐6, ICL). The environmental conditions within the controlled environment chamber were maintained as follows: photoperiod = 16 hr light (400 µmol/m^2^s^−1^ at bench level): 8h dark; air temperature = 21°C: 16°C, relative humidity = 60%. All experiments were carried out on the new fully expanded fifth leaf.

**TABLE 1:** A list of the *Triticum* genotypes used in this study, encompassing a range of ploidy levels and domestication status

Species name	Accession number/cultivar	Ploidy level	Domestication status
*Triticum baeoticum*	TRI 18344	2n	Wild
*Triticum urartu*	TRI 6735	2n	Wild
*Triticum monococcum*	TRI 28870	2n	Domesticated
*Triticum araraticum*	TRI 18513	4n	Wild
*Triticum dicoccoides*	TRI 18505	4n	Wild
*Triticum dicoccon*	TRI 16877	4n	Domesticated
*Triticum durum*	Anvergur	4n	Domesticated
*Triticum durum*	Voilur	4n	Domesticated
*Triticum aestivum*	Cougar	6n	Domesticated
*Triticum aestivum*	Crusoe	6n	Domesticated
*Triticum aestivum*	Shango	6n	Domesticated

### 2.2 Confocal imaging

The protocol used for confocal imaging was adapted from one designed for imaging *Arabidopsis thaliana* (Dow et al., 2017; Wuyts et al., 2010). Once leaf five was fully expanded, three sections approximating 1cm^2^ in the area were excised from the middle third of the leaf blade and immediately fixed in 3:1 ethanol: acetic anhydride (v/v). After fixation for 48 hr, samples were washed first in 50% ethanol, before being transferred to 70% ethanol for storage. When required, samples were removed from ethanol and subjected to a chloroform treatment to remove epicuticular wax. This was followed by progressive rehydration, bleaching, and starch digestion as per Wuyts et al., (2010). Sample staining differed slightly from Wuyts et al., (2010) in that stain time in pseudo‐Schiff propidium iodide solution was reduced from 6 to 4 hr. The clearing and mounting of leaf sections were carried out as in Wuyts et al., (2010). Once mounted and sealed, samples were imaged within two days and kept in the dark to avoid the potential for shrinkage and bleaching, respectively.

Samples were imaged using an Olympus FLUOVIEW FV1000 confocal system, and the propidium iodide fluorophore was excited using the 561 nm HeNe561 diode laser. The objective used was a 40x oil immersion objective (UPlanApo 40x, NA: 1.0). Scans were carried out at a resolution of 640 × 640 pixels, with a pixel dwell‐time of 12 µs/pixel. Scans were performed in a bidirectional manner, and no averaging was applied. In order for improved segmentation, stack Z‐step size was set at 0.3 µm. The middle layer of mesophyll cells only was imaged, due to working distance limitations of the objective and reduced fluorescence signal with leaf depth.

### 2.3 Segmentation of mesophyll cells using LithographX software

3D reconstruction and segmentation of individual mesophyll cells were carried out using the open‐source software LithoGraphX (LGX – www.lithographx.com); a spin‐off of MorphographX (Barbier de Reuille et al., 2015). Image stacks were subjected to the autoscale filter before processing using both the sieve algorithm (sieve size: 100 µm^2^) and the Gaussian blur filter (x, y, and z radius each set at 0.5 µm). Segmentation of the filtered stack was carried out using the ITK Autoseeded Watershed function within LGX (threshold: 1500). After this, seeding accuracy was examined and mistakes made during automatic segmentation (i.e., over‐ or undersegmentation of cells) were corrected manually where necessary. Cells that were not completely in the field of view or fully seeded were removed, and over‐segmented cells were reseeded as appropriate. Labeled intercellular airspaces, guard cells, and epidermal cells were also removed from the stack to leave only segmented mesophyll cells. At this point, a mesh was created using the 3D marching cubes algorithm (cube spacing: 1 µm, smooth passes: 3) and a heat map was generated to quantify cell surface area and volume paired to each individual mesophyll cell label. In order to measure cell lobing, longitudinal sections were taken through individual mesophyll cells using clipping planes within LGX, and the number of lobes per cell was counted. Geometrical information was quantified for a minimum of six individual cells from leaves of three different plants per line/ a minimum of 39 individual cells across three different plants per line.

### 2.4 Histology

Longitudinal sections (8–10 µm) of wheat leaves of varying ploidy were cut from tissue blocks fixed in ethanol/acetic acid and embedded in Technovit 7100 prior to sectioning on a Leica 2145 microtome. Sections were stained with safranin red (0.05% v/v) and mounted in Depex before imaging via light microscopy (Olympus BX51 with DP71 camera; Olympus).

### 2.5 X‐ray computed microtomography (CT) imaging and analysis

CT scans were carried out as per the protocol in Lundgren et al. (2019). Sections circa 1 cm^2^ in size were excised from the leaf blade avoiding the midrib and mounted between two plugs of polystyrene at a 45° angle to prevent sample movement during image acquisition—maximizing image quality. Each sample was scanned using a GE phoenix nanotom X‐ray µCT scanner (Waygate Technologies). Scan resolution was set at 2.75 µm, with an X‐ray voltage of 65 kV and a current of 140 µA, collecting 2400 projections using a detector exposure time of 750 ms. Scan time was 30 min. 2D projections were reconstructed into 3D volumes using a filtered back‐projection algorithm (Datos|X software, Waygate Technologies) prior to rendering and conversion to stacks of Tiff images (VG Studio Max, Volume Graphics). Masks were created to separate the sample from the background (Avizo, FEI) before stacks were cropped to a region of interest avoiding major veins and any mechanically damaged areas. Once stacks had been aligned and cropped, they were thresholded in FIJI (Schindelin et al., 2012) using the IsoData function alongside minimum algorithms to extract stacks of images representing the mask and plant tissue only. Both mesophyll porosity (% airspace/volume) and *SA_mes_*/*V_mes_* (mesophyll surface area exposed to intercellular airspace per unit leaf volume) data were extracted from these stacks using the ImageJ Particle Analyzer plugin in FIJI. Unlike the parameter *S*_mes_ (exposed mesophyll surface area per unit leaf area), expressing the exposed mesophyll surface area per volume takes into account differences in leaf or mesophyll thickness.

### 2.6 Stomatal density

Impressions of both the abaxial and adaxial leaf epidermis were collected using dental putty (Coltene Whaledent) to create negative casts. From these, positive impressions of the leaf surface were made via the application of two layers of clear nail varnish, which were mounted onto slides under a coverslip.

Counts were carried out via light microscopy on areas of the leaf on either side of a major vein (Nikon Labophot; Nikon). Each stomatal complex within the field of view was counted and stomatal density was scaled to 1 mm^2^ for each epidermis. Total mean stomatal density was calculated by summing the means of both sides of the leaf.

### 2.7 Stomatal and epidermal cell size

Stomatal size and epidermal cell size were measured on unused sections that were prepared for confocal imaging. Images were taken for both ab‐ and adaxial surfaces of the leaf blade (Olympus BX51 with DP71 camera; Olympus). Guard cell length, stomatal complex width, and stomatal complex area were all measured using FIJI software (Schindelin et al. 2012). Epidermal cell length and width were measured in the same manner.

### 2.8 Gas exchange analysis

Gas exchange measurements were carried out using an LI‐COR 6800 (LI‐6800) portable photosynthesis system (LI‐COR Biosciences, Lincoln), using an attached Multiphase Flash Fluorometer (6800‐01A) as a light source. Measurements were recorded at steady‐state, with cuvette conditions closely mirrored to those of the controlled environment chamber. The temperature exchanger was set at 21°C, and a light intensity of 425 µmol/m^2^s^−1^ was used. Relative humidity was controlled to 60%. CO_2_R was set at 400 µmol/mol and the leaf fan and flow rate were 10,000 rpm and 300 µmol/s, respectively.

All gas exchange measurements were carried out on the new fully expanded fifth leaf on the main tiller of well‐watered plants, ensuring that all plants were measured at the same developmental stage. The leaf blade was clamped before being allowed to acclimate to cuvette conditions for a minimum of 20 min—until assimilation (*A*) and stomatal conductance (*g*_s_) values were stable. Once stability was reached, datapoints were logged every 10 s for a period of 10 min and mean A and *g*_s_ values over this time were calculated. iWUE values were estimated by dividing assimilation rate by stomatal conductance (*A*/*g*_s_).

### 2.9 Statistical analysis

All statistical analysis was carried out using GraphPad Prism 8 (Graphpad Software Inc). A *p* value of <.05 was used to determine significance.

## 3 RESULTS

### 3.1 3D analysis of wheat mesophyll cells reveals changes in geometry and size linked to ploidy level

**FIGURE 1:**
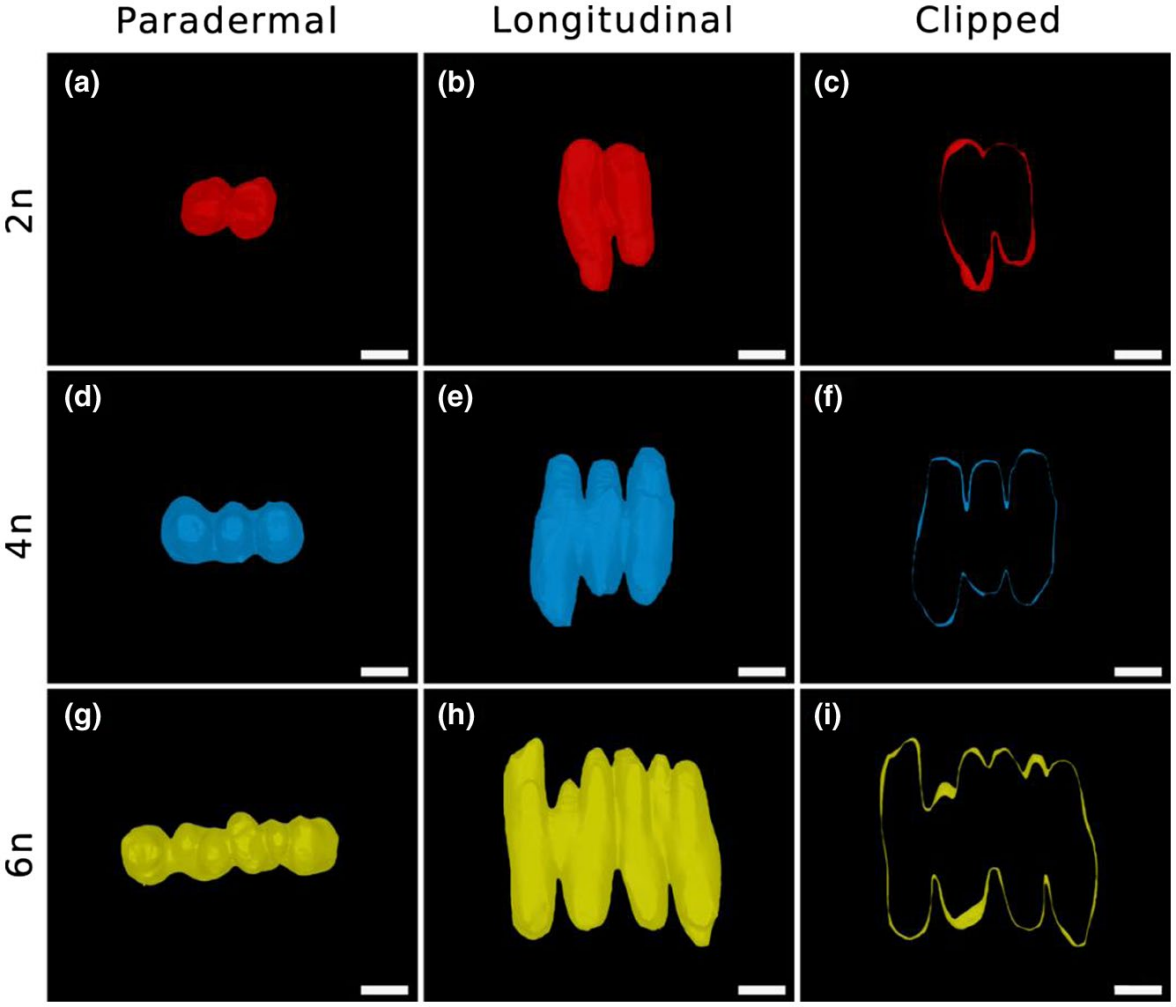
3D imaging of wheat mesophyll cells of different ploidy levels. Images of representative 2n (a–c), 4n (d–f), and 6n (g–i) mesophyll cells from a paradermal (a, d and g) or longitudinal (b, e and h) view of the leaf. (c, f and i) Show clipped images of the cells shown in (b, e and h) to highlight lobing. Cells have been false‐colored. Scale bars = 20 μm

**FIGURE 2:**
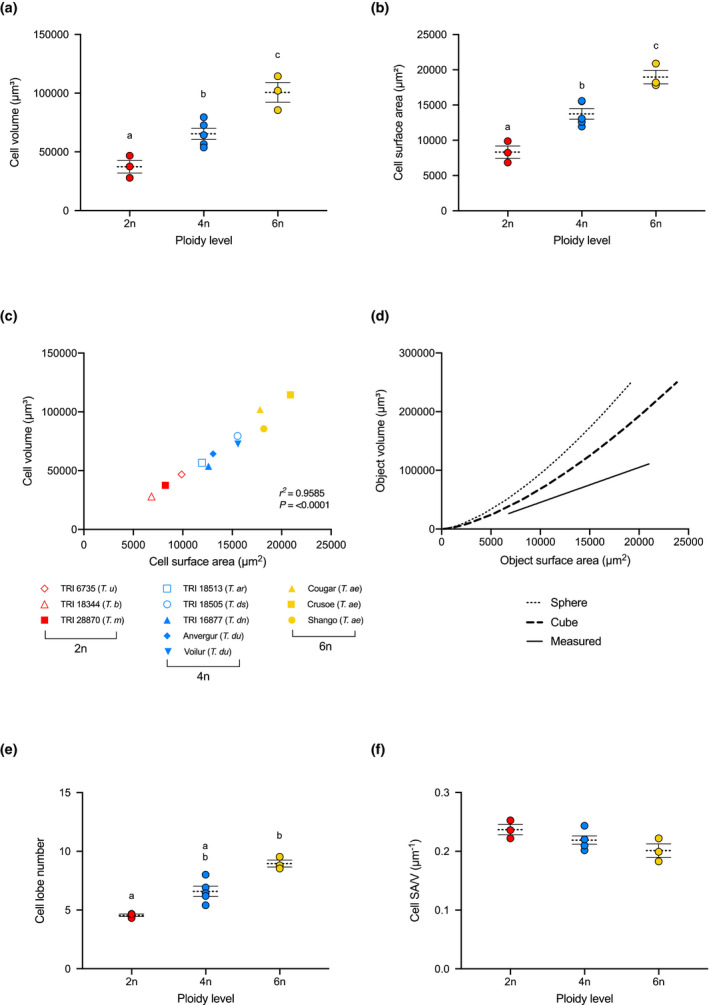
Cell volume, surface area, and lobe number in 2n, 4n, and 6n wheat cells. (a) Volume and (b) Surface Area of 2n (red), 4n (blue), and 6n (yellow) mesophyll cells. Each point represents the mean value obtained from at least 34 individual cells per line, with 3 independent leaves analyzed (lowest n per biological rep = 6), for 3 lines of 2n and 6n, and 5 lines of 4n wheat. Overall mean and standard errors are indicated for each ploidy level. Ploidy levels indicated by different letters within each plot can be distinguished from each other at the *p* = .05 confidence level (ANOVA followed by Tukey HSD test). (c) A plot of mean cell volume against mean cell surface area for individual 2n, 4n, and 6n lines (as indicated in the key). A linear correlation analysis indicated a strong positive correlation (*r^2^* = .9585, *p* < .0001). (d) The expected curvilinear relationships between surface area and volume for a sphere (dotted) and a cube (dashed line) in comparison to a linear relationship (solid line). (e) Cell lobe number plotted against ploidy level for the samples, with analysis as described in (a). (f) Cell Surface Area to Volume ratio (SA/V) plotted against ploidy level, with analysis as described in (a). The samples of different ploidy levels cannot be distinguished from each other at the *p* = .05 confidence level

To image wheat mesophyll cells, leaf samples from a variety of *Triticum* lines (2n, 4n, 6n—listed in Table 1) were processed for confocal microscopy, as described in the Materials and Methods. Confocal stacks were exported into LithoGraphX to enable the segmentation of the image stacks, generating 3D representations of individual mesophyll cells (Figure 1). These data can be compared with corresponding images of wheat mesophyll cells obtained using stained tissue sections in which individual cells have been highlighted (Figure S1). Clearly, the confocal‐derived data provide a fuller picture of mesophyll cell shape for all ploidy levels. An overview of the process, pipeline for extracting 3D geometry from confocal stacks is provided in Figure S2. Quantitative analysis of data derived from the 3D representations of cells from 11 wheat genotypes varying in ploidy level is shown in Figure 2.

Our analysis revealed clear visual differences in mesophyll cell size and shape‐dependent upon the ploidy level of the plant from which the leaves were obtained (Figure 1a,d,g). Cells of the three ploidy levels could be distinguished from each other by volume (ANOVA, *F*(2, 8) = 22.81, *p* = .0005) with the 2n cells being the smallest and 6n cells the largest (Figure 2a). Wheat mesophyll cells are lobed in nature (Dunstone & Evans, 1974; Harwood et al., 2020; Jung & Wernicke, 1990; Parker & Ford, 1982) and our data confirmed this observation in wheat of all three ploidy levels (Figure 1a,d,g), As cell volume increased, so did the number of lobes, with lobe number ranging from 2 to 11 in 2n cells (median = 4, mean = 4.32), 3–15 in 4n cells (median = 6, mean = 6.59) and 4–18 in 6n cells (median = 8, mean = 8.96) (Figure 2e). This lobing of the mesophyll cells was apparent both in paradermal views (Figure 1a,d,g) and in longitudinal views of the leaf (Figure 1b,e,h), particularly when the longitudinal views were clipped to highlight the cell boundaries (Figure 1c,f,i). These views of the mesophyll cells from different aspects highlighted the fact that lobing in wheat mesophyll cells is complex, involving both paired partial indentations along the cell long‐axis (parallel to the leaf long‐axis) to produce a series of main lobes, but also differential growth of these lobes along the axis of the leaf depth (z‐axis) to generate tooth‐like projections, both toward the abaxial and adaxial sides of the leaf surface. Measurement of individual lobe width along the cell long‐axis in 2n, 4n, and 6n cells did not reveal any significant difference in lobe size (Figure S3) (ANOVA, *F*(2, 8) = 3.645, *p* = .0749, *n* = 3).

To investigate this potentially complex interaction of changes in cell volume and shape across the different ploidy levels, we looked at the relationship of cell volume and surface area. As expected, the increase in cell volume observed as ploidy level increased from 2n to 4n to 6n was reflected by an increase in cell surface area (ANOVA, *F*(2, 8) = 31.12, *p* =.0002, *n* = 3) (Figure 2b). When cell volume was plotted against cell surface area a positive relationship was observed (Figure 2c). Interestingly, this relationship was close to linear (Pearson correlation analysis, *r^2^* = .959, *P* < .0001, *n* = 610). If cells undergo isometric growth, the surface area of a cell is expected to increase by the square of linear dimension, whereas volume is expected to increase according to a cubic relationship, that is, isometric growth results in a non‐linear relationship of cell volume and surface area (as depicted in Figure 2d). Analysis of cell surface area to volume ratio (SA/V) for cells of different ploidy levels revealed that although there was a slight decline in SA/V for 6n cells relative to cells of 4n or 2n ploidy, the cells of different ploidy levels could not be distinguished from each other based on this parameter (ANOVA, *F*(2, 8) = 3.388, *p* = .0859, *n* = 3). Taken together with the observational data presented in Figure 1, our data are consistent with the hypothesis that an increase in mesophyll cell volume has occurred during the evolution of 2n, 4n, and 6n wheat but that this has incurred anisotropic growth, leading to a change in cell shape which maintains a relatively high surface area to volume ratio at the level of the mesophyll cell.

### 3.2 Altered geometry is linked to altered cell packing and leaf mesophyll architecture

To investigate how the observed changes in mesophyll cell size and shape related to the overall internal architecture of the leaf, we performed a series of microCT scans on selected wheat lines (TRI 28870, TRI 16877, Anvergur & Voilur). These augmented data from a previous microCT analysis of wheat lines (Lundgren et al., 2019), providing a library of CT data with which to investigate the relationship of cell‐level size and shape with tissue‐level structural parameters (exemplar 3D reconstruction is shown in Figure S4).

**FIGURE 3:**
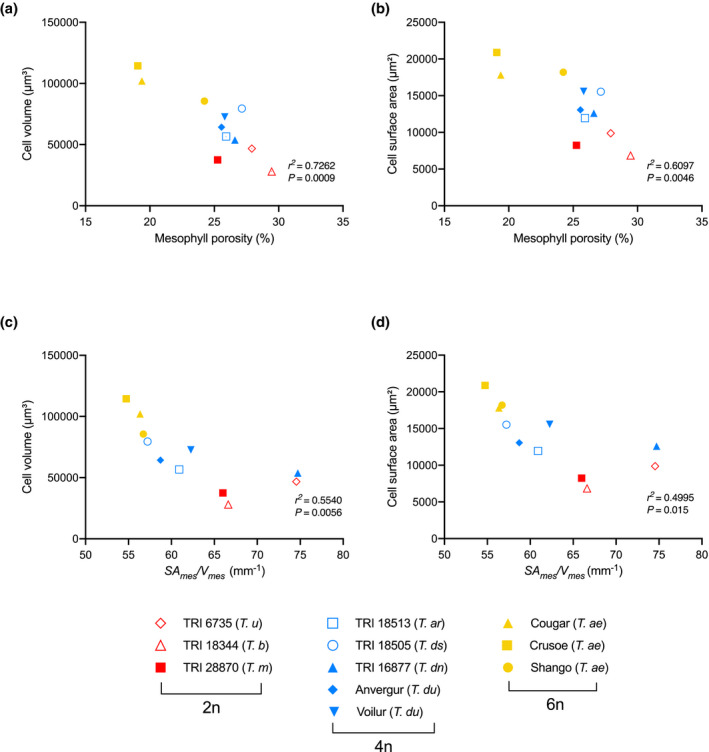
The relationship of mesophyll cell size and surface area to tissue level porosity and exposed surface area with ploidy. (a) Mean mesophyll cell volume and (b) Mean cell surface area for 2n (red), 4n (blue), and 6n (yellow) wheat lines plotted against mean mesophyll porosity. Linear correlation analysis (Pearson) indicated a negative relationship between cell volume and tissue porosity (*r^2^* = .726, *p* = .0009) and cell surface area and tissue porosity (*r^2^* = .610, *p* = .0046). (c) Mean mesophyll cell volume and (d) Mean mesophyll cell surface area for 2n (red), 4n (blue), and 6n (yellow) wheat lines plotted against mean exposed mesophyll surface area (*SA_mes_*/*V_mes_*). Linear correlation analysis (Pearson) indicated a negative relationship between cell volume and *SA_mes_*/*V_mes_* (*r^2^* = .554, *p* = .0066) and cell surface area and *SA_mes_*/*V_mes_* (*r* ^2^ = .500, *p* = .015). The key to individual 2n, 4n, and 6n lines analyzed is shown. Values for mesophyll porosity for *T*. *u*, *T*. *b*., *T*. *ar*, *T*. *ds*, Cougar, Crusoe, and Shango are taken from Lundgren et al. (2019)

One key parameter obtained from microCT analysis is porosity, a measure of the relative amount of airspace within a leaf to the total volume of the leaf. When mean mesophyll cell volume for leaves of different ploidy levels was plotted against the mean porosity of these leaves, an inverse relationship was revealed (Pearson correlation, *r^2^* = .762, *p* = .0009) (Figure 3a). Thus, the increase in cell volume observed at higher ploidy levels (Figure 2a) has been accompanied by an increase in leaf density (a decrease in the relative amount of airspace). When mesophyll cell surface area was plotted against leaf mesophyll porosity (Figure 3b) a comparable inverse relationship was observed (Pearson correlation, *r^2^* = .61, *p* = .0046), indicating that as leaves have become denser during the evolution of polyploid wheat, the potential surface area for gas exchange within the leaf across the mesophyll surface at the cellular level has increased. The actual exposed mesophyll surface area for gas exchange per unit volume of tissue (*SA_mes_*/*V_mes_*) was calculated from the CT data and plotted against individual mesophyll cell volume (Figure 3c). As cell volume increased there was a trend for decreasing *SA_mes_*/*V_mes_* (Pearson correlation, *r* ^2^ = .554, *p* = .0056), although the visual inspection of the data suggests that this may not be a linear relationship across the entire spectrum of *SA_mes_*/*V_mes_*. Similarly, when *SA_mes_*/*V_mes_* was plotted against mesophyll cell surface area (Figure 3d) there was a negative relationship (Pearson correlation, *r* ^2^ = .500, *p* = .0015), again with the proviso that this may not be a linear relationship toward higher values of *SA_mes_*/*V_mes_*. Taken into context with the data shown in Figure 2, the results indicated that despite the changes in cell geometry (reflecting anisotropic growth) to maintain a relatively constant amount of surface area to volume at the level of the individual cell, the actual total integrated exposed mesophyll surface area per volume available for gas exchange at the tissue level within the leaf (*SA_mes_*/*V_mes_*) has actually decreased as ploidy level has increased.

The only way around this apparent conundrum is for the packing of mesophyll cells to have altered, with the degree of cell separation that occurs during mesophyll differentiation adjusting so that in leaves with larger, more lobed cells (e.g., hexaploid, 6n), the mesophyll cells have remained relatively more conjoined than in lower ploidy leaves comprising smaller, less lobed cells. This would also account for the measured decrease in airspace (lower porosity) of the higher ploidy level leaves (Figure 3a), leading to an overall denser mesophyll consisting of more closely packed, more lobed cells.

**FIGURE 4:**
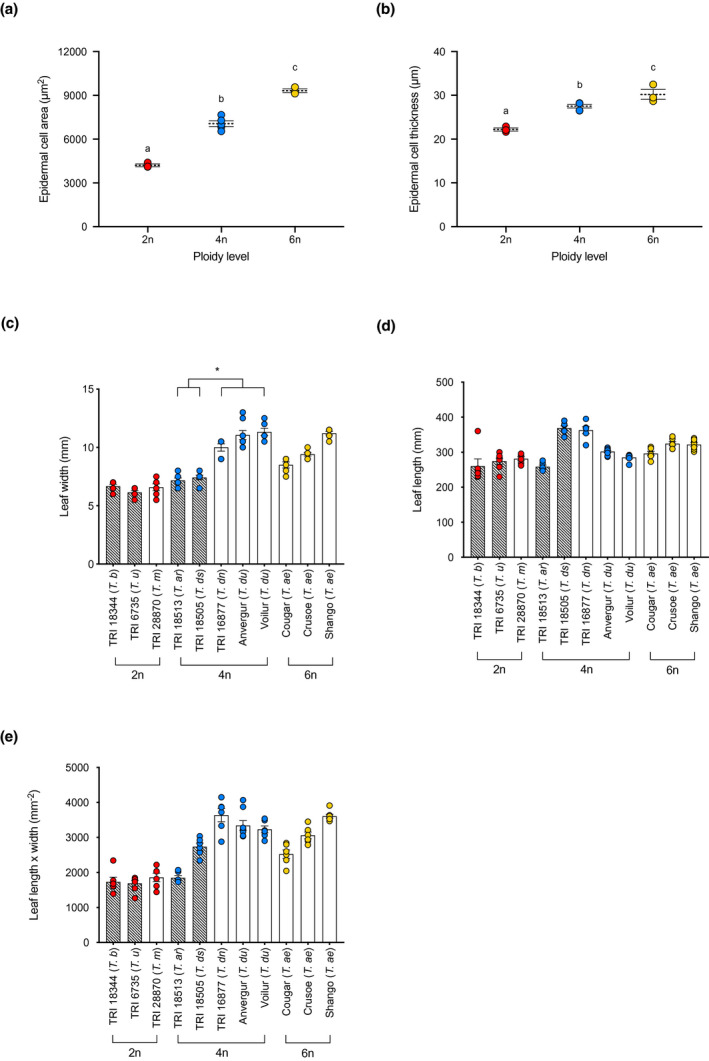
Epidermal cell size and leaf area in wheat leaves of different ploidy. (a) Epidermal cell area and (b) thickness of 2n (red), 4n (blue), and 6n (yellow) mesophyll cells. Each point represents the mean value obtained from at least 50 cells, with a minimum of 5 independent leaves analyzed, for 3 lines of 2n and 6n, and 5 lines of 4n wheat. Overall mean and standard errors are indicated for each ploidy level. Ploidy levels indicated by different letters within each plot can be distinguished from each other at the *p* = .05 confidence level (ANOVA followed by Tukey HSD test). (c) Mean leaf width, (d) leaf length, and (e) leaf length x width for individual wheat species or cultivars of 2n (red), 4n (blue), and 6n (yellow) ploidy level. Data points represent the analysis of individual leaves, with bars indicating mean values with standard error. Hatched bars indicate non‐domesticated lines, open bars domesticated lines. Within the ploidy level, comparisons were made using an unpaired two‐tailed *t* test, with an asterisk indicating that the within ploidy groups marked could be distinguished at the *p* = .05 confidence level

With respect to the epidermis, our analysis indicated that an increase in epidermal cell size has also occurred during the evolution of 6n wheat. There has been a step‐wise increase in both mean epidermal cell length and cell width from 2n to 4n to 6n wheat, leading to an increase in epidermal cell area (Figure 4a), and thickness (Figure 4b). However, epidermal cell shape has remained similar at all ploidy levels. When gross leaf width and length were measured, there was a trend for increased leaf area (approximated by multiplying width and length) as ploidy increased (Figure 4e). This reflected increases in both width and length of the leaves, although length was not found to significantly differ with ploidy (Figure 4d). Interestingly, within the 4n lines there appeared to be a difference between the domesticated and non‐domesticated cultivars, with the leaves of domesticated 4n wheat tending to be wider than the non‐domesticated Figure 4c (unpaired *t* test, *t*(3) = 6.573, *p* = .0072, *n* > 4 per line) with a larger leaf area (Figure 4e) (unpaired *t* test, *t*(3) = 3.038, *p* = .0560, *n* > 4 per line). This trend was not seen when comparing the epidermal cell widths in domesticated and non‐domesticated 4n lines, suggesting that there has been an extended round of anticlinal cell divisions in the epidermis of domesticated 4n lines compared to the non‐domesticated lines. Overall, an increase in mean leaf width distinguished the domesticated (4n and 6n lines) from the non‐domesticated (4n and 2n lines) (unpaired *t* test, t(9), *p* = .0116, *n* > 4 per line), though the domesticated 2n line *T. monococcum* was an exception in this analysis.

### 3.3 Domestication of tetraploid wheat has been accompanied by improved water‐use efficiency, iWUE

**FIGURE 5:**
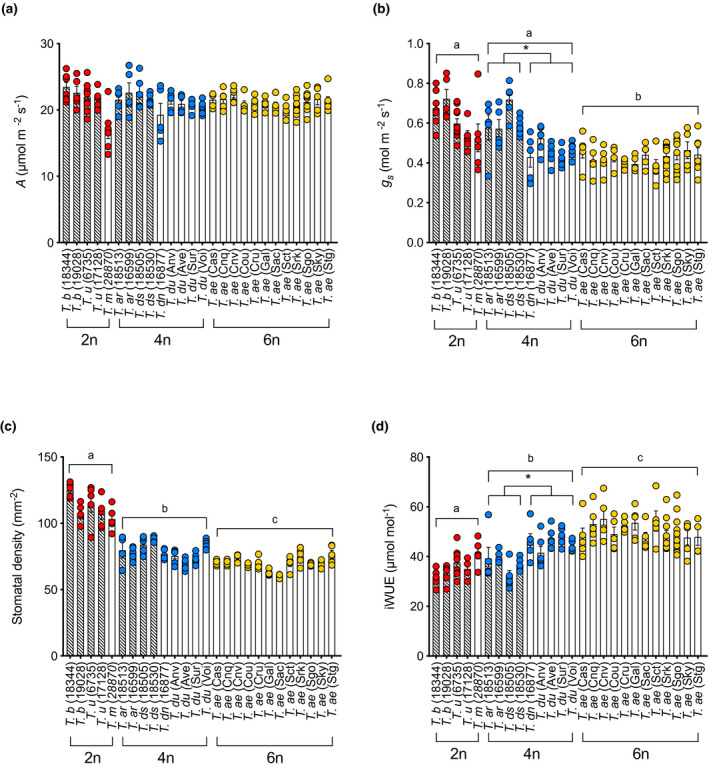
Changes in water‐use efficiency between and within wheat lines of different ploidy. (a) CO_2_ assimilation rate, *A*, (b) Stomatal conductance, *g*_s_, (c) stomatal density, and (d) instantaneous water‐use efficiency, iWUE, for a range of species/cultivars of 2n (red), 4n (blue), and 6n (yellow) wheat. Each data point represents the analysis of an independent leaf, with bars indicating the mean value with standard error. Between the ploidy level, comparisons were performed using a one‐way ANOVA, followed by Tukey's HSD, with groups that can be distinguished from each other at the *p* = .05 confidence limit indicated by different letters. Within the ploidy level, comparisons were performed using an unpaired two tailed *t* test, with an asterisk indicating that the groups marked can be distinguished from each other at the *p* = .05 confidence limit

Our analysis of mesophyll cell size, shape, and packing indicated major changes have occurred during the evolution of polyploid wheat. To investigate whether these changes in leaf cell architecture correlated with altered leaf function in terms of carbon assimilation or water loss, we performed a series of gas exchange analyses. This included a larger panel of *Triticum* genotypes as part of a wider screen of physiological performance—additional lines summarized in Table S1. Measurement of CO_2_ assimilation rate under ambient growth conditions (*A*) indicated no significant difference in photosynthetic performance dependent on ploidy level (ANOVA, *F*(2, 23) = 0.1703, *p* = .8445, *n* > 4 per line) (Figure 5a), confirming and extending previous analyses (Lundgren et al., 2019). Analysis of photosynthetic capacity (*A*_max_) supported results of previous investigations (Austin et al., 1982; Jellings & Leech, 1984), with maximum rates of photosynthesis being higher in 2n plants than 6n plants, while 4n plants were intermediate (Figure S5). In contrast, analysis of stomatal conductance, *g*_s_, indicated a significantly lower value in the 6n lines compared with the 4n and 2n lines (Figure 5b). Interestingly, however, the domesticated 4n lines could be distinguished from the non‐domesticated 4n lines based on *g*_s_ (unpaired *t* test, *t*(7) = 4.254, *p* = .0001, *n* > 4), with the domesticated 4n lines having relatively low *g*_s_ values (but still slightly higher than those observed in the domesticated 6n lines) (unpaired *t* test, *t*(15) = 1.980, *p* = .0664, *n* > 4 per line), and the non‐domesticated 4n lines having *g*_s_ values indistinguishable to those measured in the 2n lines (unpaired *t* test, *t*(7) = 0.06527, *p* = .9498, *n* > 4 per line).

To investigate whether the differences in *g*_s_ observed in the 4n wheat lines might be linked to differences in stomatal size and density we measured these parameters across all the wheat lines investigated in this report (Figure 5c, Figure S6). Consistent with previous data (Lundgren et al., 2019), 2n, 4n, and 6n lines could be distinguished from each other based on stomatal density (ANOVA, *F*(2, 23) = 87.73, *p* = .0001, *n* > 4 per line), with higher ploidy leaves having a lower stomatal density. Importantly, there was no indication of any change in stomatal density between domesticated and non‐domesticated 4n lines which might account for the differences in *g*_s_ observed in Figure 5b. Similarly, analysis of stomatal size confirmed and extended previous data that an increase in ploidy in wheat has been accompanied by an increase in stomatal size (measured via guard cell length, stomatal complex width or area) (Figure S6). Again, comparison of the domesticated and non‐domesticated 4n lines with respect to the stomatal size did not reveal any differences which might account for the measured differences in *g*_s_. Returning to the analysis of mesophyll cell size and shape described in Figures 1 and 2, there was no apparent difference in these parameters that allowed domesticated and non‐domesticated 4n lines to be easily distinguished.

Instantaneous water‐use efficiency (iWUE) is a measure of the amount of water lost by a leaf per amount of carbon fixed by photosynthesis and is derived from the ratio of carbon assimilation rate, *A*, and stomatal conductance, *g*_s_. As shown in Figure 5a, assimilation rates were similar across all ploidy levels, whereas there were clear differences in *g*_s_ (Figure 5b). Consequently, extending previous data, 6n lines had a significantly higher iWUE than 4n or 2n wheat lines (ANOVA, *F*(2, 23) = 26.16, *p* = .0001, *n* > 4 per line) (Figure 5d). Interestingly, our analysis of the range of 4n lines showed that domesticated 4n lines had a significantly higher iWUE than the non‐domesticated 4n lines (unpaired *t* test, *t*(7) = 4.035, *p* = .005, *n* > 4 per line).

## 4 DISCUSSION

### 4.1 Imaging wheat mesophyll cells in 3D

Previous analyses of wheat mesophyll cell size and shape have depended on 2D imaging of what are, by their very nature, 3D objects. Although well established, such 2D analytical methods become challenging when faced with more complex, non‐symmetrical shapes. Recent analyses have suggested that even in these situations 2D processing approaches may under‐estimate actual values of, for example, cell volumes and surface areas (e.g., Harwood et al., 2020; Mathers et al., 2018; Théroux‐Rancourt et al., 2017). Indeed, Théroux‐Rancourt et al., 2017, provide a detailed summary of contributing factors to sources of error in estimating leaf 3D geometry from 2D image data. In this respect the confocal microscopy approach described here, linked to the more advanced image segmentation and analytical tools now available, provides a powerful tool to gain new insight into leaf cell size and shape in 3D (Barbier de Ruielle et al., 2015; Earles, Theroux‐Rancourt, et al., 2018; Wuyts et al., 2010). Of course, all imaging approaches have their limitations. For example, with the CT method used here, resolution is 2.75 µm, so that intercellular airspaces of this order of size or smaller will not be accurately detected, leading to errors in the absolute values of both porosity and, linked to this, *SA_mes_*/*V_mes_*. These errors are likely to be similar for all the species examined here (which have the same basic leaf structure), so we are confident that the relative shifts in, for example, porosity between ploidy levels are correct which, taken into account with the large changes in cell volume/surface area observed, gives confidence to our conclusions. Nevertheless, the unavoidable trade‐off between volume of tissue observed and resolution means that absolute values of airspace should be viewed as estimates of the real values. Moreover, the relatively large volume of sample analyzed here, in combination with the fact that cells remain in situ, means that one can gain an easier understanding of how cells are arranged in space, providing insight into how these patterns may have arisen, and the potential consequences of these patterns on leaf form and function (Dow et al., 2017). The combined imaging approach reported here, integrating segmented confocal data with CT imaging gives new insights into wheat leaf structure.

### 4.2 The evolution of wheat cell geometry and leaf structure

Previous work reported that hexaploid wheat mesophyll cells are larger and have more complex shapes than those from tetraploid and diploid progenitors (Dunstone & Evans, 1974; Jellings & Leech, 1984; Parker & Ford, 1982; Pyke et al., 1990). Our research advances this work by providing a more accurate quantitative analysis across a broader spectrum of wheat species differing in ploidy level, providing new insight into the evolutionary trajectory of cell shape change. The results indicate that the evolution of cells of higher ploidy has been accompanied by an increase in cell volume, substantiating the general trend observed in many other plant species (Beaulieu et al., 2008; Katagiri et al., 2016; Kondorosi et al., 2000; Sugiyama, 2005) (although exceptions to this rule do exist (Tsukaya, 2008, 2013)). However, our data suggest that this increase in cell size has not occurred by the simple isometric enlargement of an original 2n mesophyll cell shape. Rather, as cells have become larger (presumably driven by the increase in ploidy level), cell shape has accommodated to maintain a relatively higher than expected surface area to volume ratio. At first sight, this could be interpreted on biophysical/biochemical grounds: as a cell becomes larger its metabolic requirements, as dictated, for example, by photosynthesis, will increase proportionally to the cube of linear dimension, yet the capacity for metabolite flux across the cell surface will increase only by the square of the linear dimension. Shape changes that maintain a particular surface area to volume ratio (e.g., increased lobing) could facilitate these increased metabolic requirements (Austin et al., 1982; Sage & Sage, 2009; Tholen et al., 2012). However, our finding that the actual total amount of internal mesophyll surface area available for gas exchange (*SA_mes_*/*V_mes_*) decreases in leaves containing cells of higher ploidy suggests that this could be a simplistic interpretation as it does not take into account the potential for differential degrees of cell‐cell contact as size and shape changes occur. As wheat cells have enlarged over evolutionary time this has been accompanied by a relative decrease in airspace, consistent with previous observations in arabidopsis where engineering leaves with larger cells led to denser leaves with relatively less airspace and lower *S_me_*_s_ (Dorca‐Fornell et al., 2013; Lehmeier et al., 2017). The functional interpretation of this complex balance of cell‐level and tissue‐level surface area to volume ratio is discussed further below.

With respect to the actual mechanism of shape change in wheat cells, our data are consistent with the hypothesis that the increase in mean lobe number observed in 2n, 4n, and 6n cells reflects an evolutionary process whereby a conserved mechanism of lobe formation occurs irrespective of ploidy level: individual lobe size along the cell long‐axis remains approximately constant against a background of increasing cell axis length. The most significant advances in our understanding of lobe formation in plant cells have come from the analysis of the interdigitation of epidermal cells in eudicots such as arabidopsis (Bidhendi et al., 2019; Sampathkumar et al., 2014; Sapala et al., 2019). This work has started to reveal the complex interplay of the cytoskeleton, vesicle movement, and biophysical feedbacks which allow complex yet harmonized differential cell growth to occur, generating cellular patterns. To what extent similar mechanisms underpin the differential growth responsible for wheat mesophyll lobing remains to be elucidated, although the cytoskeleton has been linked to the formation of lobes in these cells (Giannoutsou et al., 2013; Jung et al., 1993; Jung & Wernicke, 1990).

### 4.3 The influence of mesophyll cell geometry, size, and packing on photosynthesis and water‐use efficiency

It has been argued that a larger genome size (linked to higher ploidy level and larger cell size) is associated with a limitation on photosynthetic capacity (Roddy et al., 2020). In contrast to previous investigations focussing on photosynthetic capacity in wheat (Austin et al., 1982; Dunstone & Evans, 1974), under our growth conditions, we observed no difference in CO_2_ assimilation rates related to ploidy level or cell volume—although a trend of decreased *A_max_* with increased ploidy was seen. However, we did observe a significant decrease in stomatal conductance, *g*_s_, as ploidy level increased. Our data are consistent with the hypothesis that the increases in stomatal size and decreases in stomatal density that occur as epidermal cell size increases with ploidy leads to altered gas flux across the epidermis which influences mesophyll differentiation (Lundgren et al., 2019). In an evolutionary context, this has led to an overall decrease in *SA_mes_*/*V_mes_* (therefore less exposed surface area for water loss) without apparently decreasing steady‐state CO_2_ assimilation, suggesting that (at least under our conditions) *SA_mes_*/*V_mes_* was not limiting photosynthesis (although it may play a role in limiting *A_max_*). The fact that the shape change observed at the level of the individual mesophyll cell has led to an evolutionary more or less constant ratio of cell surface area to volume suggests that this altered geometry may be important for some aspect of leaf function other than CO_2_ flux. Exactly what this function remains open to speculation. For example, the relatively larger contact area between cells might facilitate the increased local flux of metabolites within the leaf, might provide more structural integrity by cell interdigitation, or may enable leaf thickness to be minimized, with knock‐on effects on light capture and distribution within the leaf (Tholen et al., 2012; Vogelmann & Evans, 2002; Xiao et al., 2016).

An interesting and unexpected finding from our analysis was the difference in *g*_s_ observed between domesticated and non‐domesticated tetraploid (4n) lines, with domesticated lines showing a relatively lower *g*_s_, more similar to modern 6n lines than the progenitor 2n lines. The mechanistic basis of this shift is unclear, with no obvious underpinning in the structural parameters measured in this investigation (stomatal size and density, mesophyll cell size, and shape, or *SA_mes_*/*V_mes_*). Future work will be focussed on expanding the range of 4n lines analyzed to confirm the universality of this observation. Even without fully understanding the mechanism, the finding that some 4n wheat lines display improved *g*_s_ suggests that this may have been an important step in the wheat selection, and may provide a route to trait selection for improved water‐use efficiency in agriculture. Similarly, our finding that increased mesophyll cell volume resulting from increased ploidy is linked to denser leaves with improved iWUE raises the question as to whether there is scope for engineering or selecting wheat with larger mesophyll cells and decreased *SA_mes_*/*V_mes_* to improve water‐use efficiency without limiting carbon assimilation in this essential crop. While our results suggest that the improved crop water use of domesticated wheat is influenced by leaf structural changes associated with changing ploidy levels, it is also possible that the improved *g*_s_ and iWUE traits observed in domesticated wheat lines may have been separately selected for during domestication. Manipulation of mesophyll cell size within ploidy level may enable further elucidation of wheat leaf structure/function relationships.

## CONFLICT OF INTEREST

The authors are not aware of any conflict of interest.

## AUTHOR CONTRIBUTIONS

MJW, MF‐S performed the experiments; CJS and SM supervised CT analysis and interpretation; MJW and AJF interpreted the results and wrote the paper, with contributions from all authors. AJF and RS designed the study and AJF led the project.

## Supporting information

Fig S1‐S6‐Table S1
