# Supplementary material for: Ploidy influences wheat mesophyll cell geometry, packing and leaf function
Source: Plant Direct. 2021 Apr 7;5(4):e00314. doi: 10.1002/pld3.314 (PMC8026107; doi:10.1002/pld3.314)
Supplement: Supplementary file 1 — Fig S1‐S6‐Table S1 [file PLD3-5-e00314-s001.pdf]

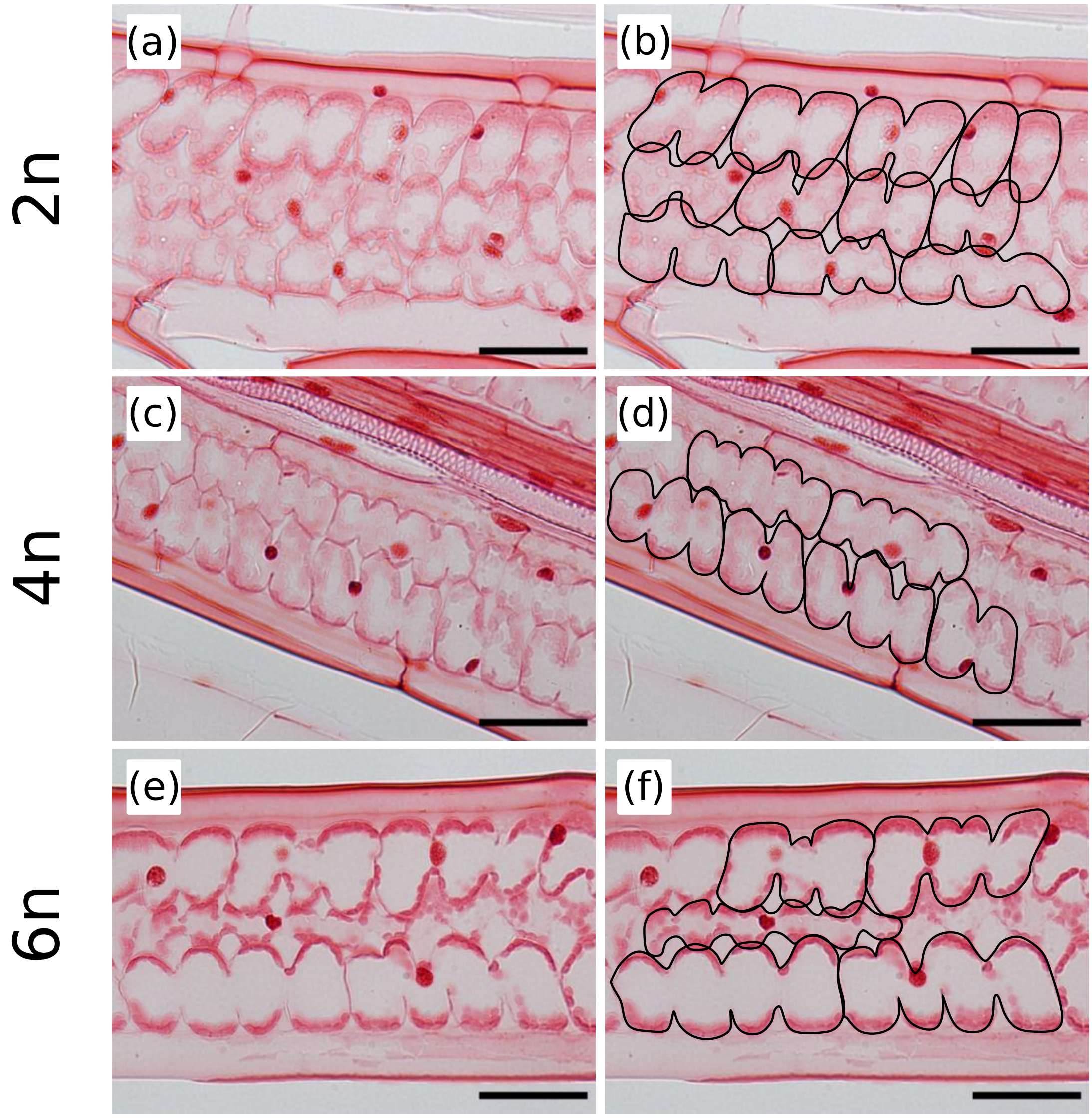

Figure S1: Pipeline for 3D image analysis of wheat mesophyll cells.
Images of representative 2n (**a,b**), 4n (**c,d**) and 6n (**e,f**) mesophyll cells in longitudinal section. Cell boundaries of complete individual mesophyll cells from (a,c,e) are outlined in (b,d,f). Scale bars = 50 µm.

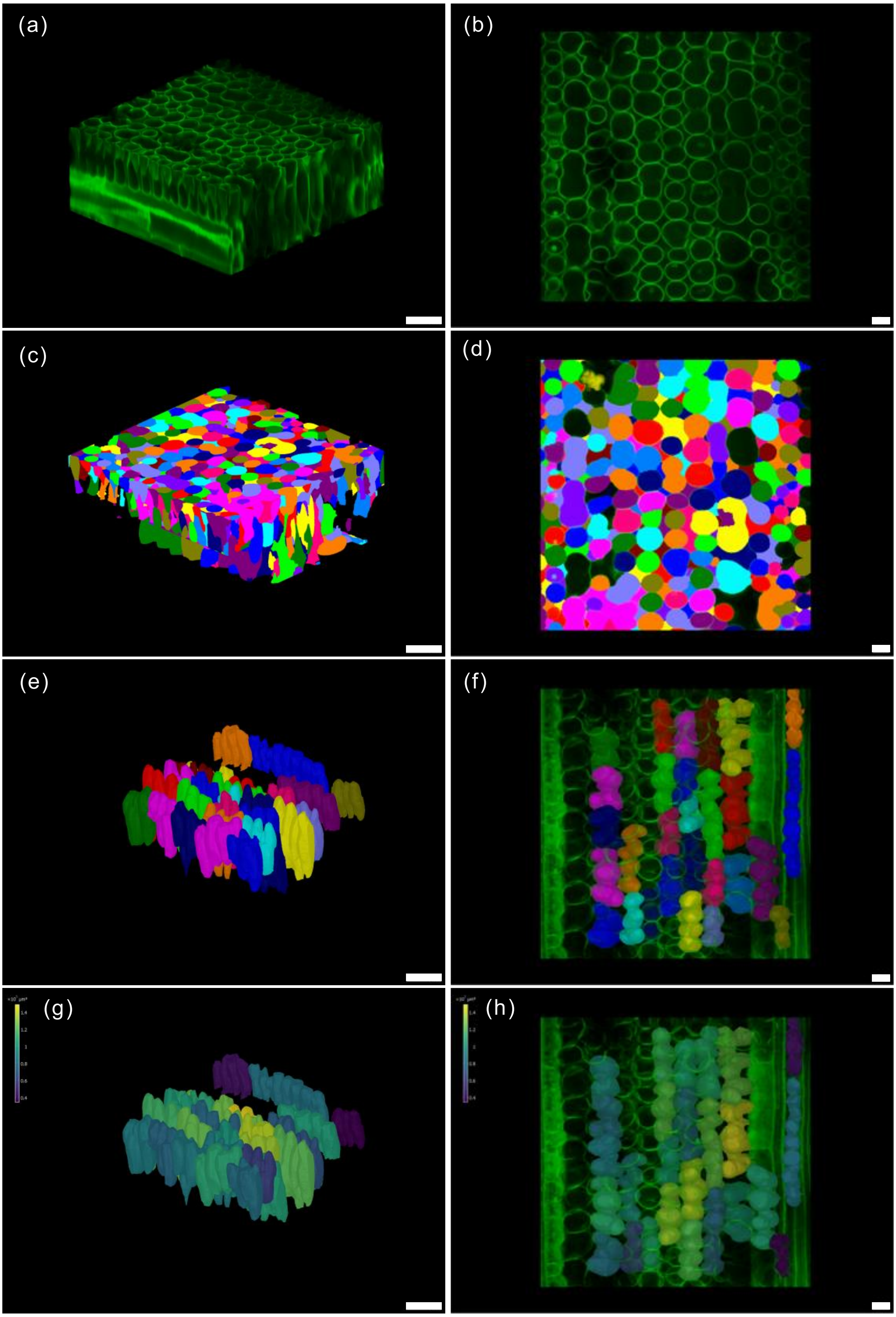

Figure S2: Pipeline for 3D image analysis of wheat mesophyll cells.
(a) A stack of individual confocal images **(b)** is segmented using LGX software. Each initial cell is randomly assigned a colour label (**c,d**) before manual adjustment to ensure veracity with original image stack. A mesh is created for each cell (**e,f**), allowing values linked to 3D geometry to be extracted, including the assignment of heat map values for, e.g., cell volume (**g**) and viewing of cell arrangement in the context of leaf anatomy (**h**). Scale bars in (a,c,e,g) = 50 µm; (b,d,f,h) = 20 µm.

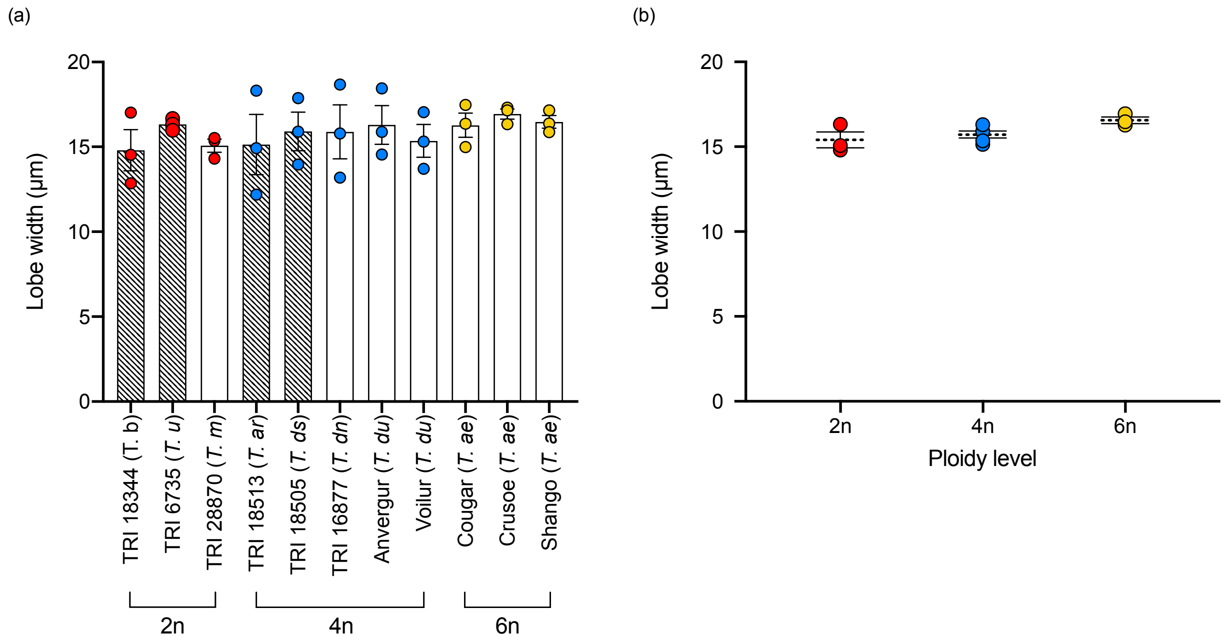

Figure S3: Lobe width of wheat mesophyll cells of different ploidy.
(**a**) Mean lobe width for individual wheat species or cultivars of 2n (red), 4n (blue) and 6n (yellow) ploidy level. Data points represent analysis of individual leaves, with bars indicating mean values with standard error. Hatched bars indicate non-domesticated lines, open bars domesticated lines. (**b**) Lobe width of 2n (red), 4n (blue) and 6n (red) mesophyll cells. Each point represents the mean value obtained from at least 34 cells, with 3 independent leaves analysed, for 3 lines of 2n and 6n, and 5 lines of 4n wheat. Overall mean and standard errors are indicated for each ploidy level. Ploidy levels indicated by different letters within each plot can be distinguished from each other at the P= 0.05 confidence level (ANOVA followed by Tukey test).

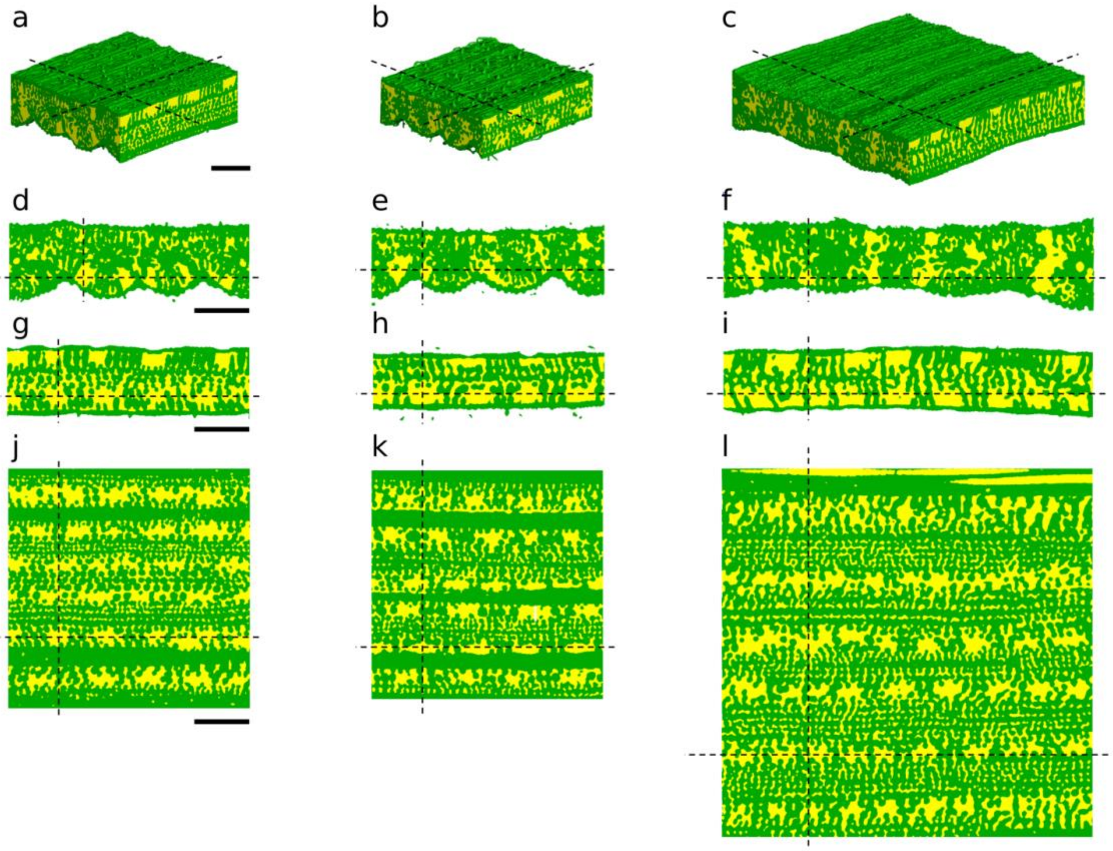

Figure S4. CT images of wheat lines of different ploidy.
3D renderings of leaf sections of (**a**) *T. monococcum* (2n), (**b**) *T. dicoccon* (4n) and (**c**) *T. durum* (4n) cv. Voilur. Dense leaf tissue is coloured green, whilst airspaces are shown in yellow. Panels (**d,e,f**) show the leaf in transverse section; (**g,h,i**) in longitudinal section and (**j,k,l)** paradermal section. Dashed lines indicate the plane from which sections have been taken. Scale bars = 200 μm.

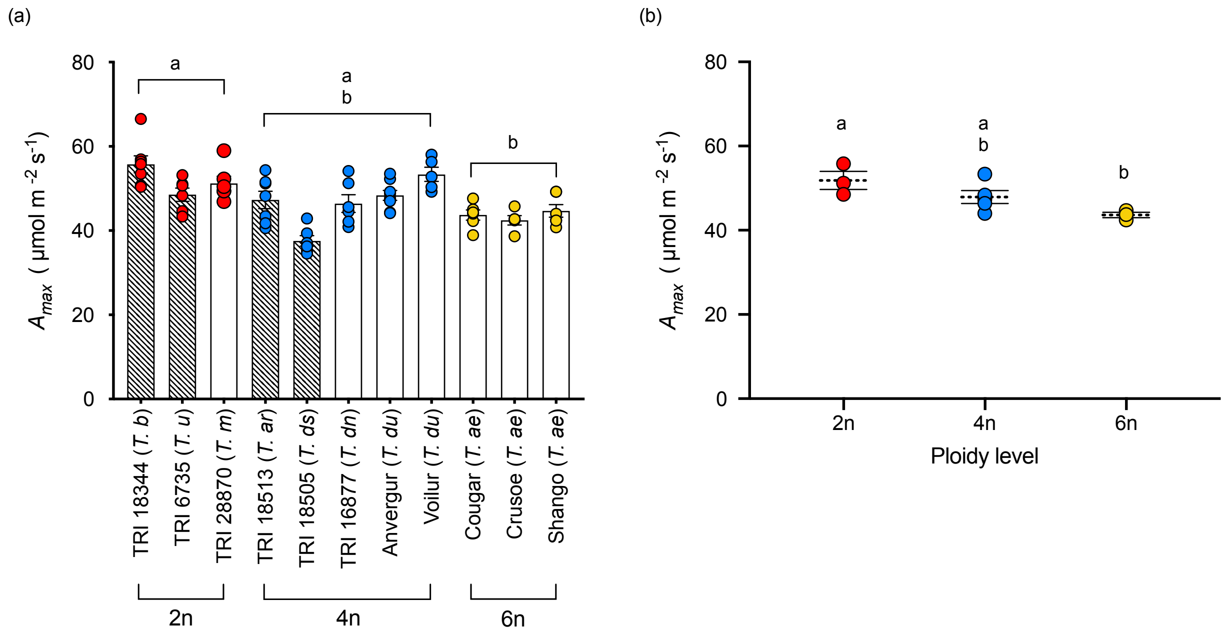

Figure S5: Photosynthetic capacity of wheat lines of different ploidy.
(**a**) Mean A_{max} for individual wheat species or cultivars of 2n (red), 4n (blue) and 6n (yellow) ploidy level. Each data point represents the analysis of an independent leaf, with bars indicating the mean value with standard error. Between ploidy level comparisons were performed using a one-way ANOVA, followed by Tukey's HSD, with groups that can be distinguished from each other at the *P*= 0.05 confidence limit indicated by different letters. Hatched bars indicate non-domesticated lines, open bars domesticated lines. (**b**) Mean A_{max} of 2n (red), 4n (blue) and 6n (red) wheat lines. Overall mean and standard errors are indicated for each ploidy level. Ploidy levels indicated by different letters within the plot can be distinguished from each other at the *P*= 0.05 confidence level (ANOVA followed by Tukey test).

.

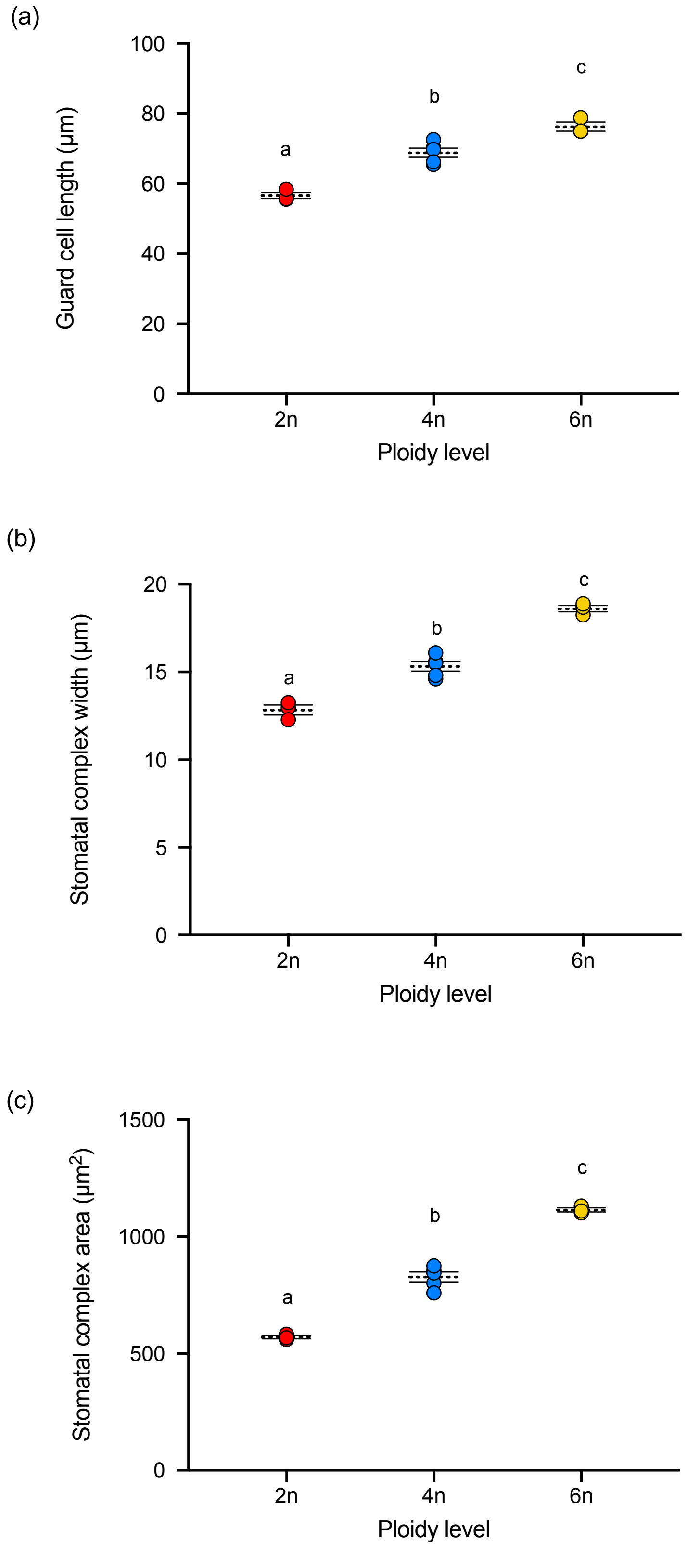

Figure S6. Stomatal size characteristics in wheat of different ploidy level.
(**a**) Guard cell length, (**b**) Stomatal complex width and (**c**) Stomatal complex area of 2n (red), 4n (blue) and 6n (red) wheat lines. Each point represents the mean value obtained from 30 stomata observed in five independent leaf samples for each of 3 species/cultivars of 2n and 6n wheat, and 5 species of 4n wheat. Overall mean and standard errors are indicated for each ploidy level. Ploidy levels indicated by different letters within each plot can be distinguished from each other at the P=0.05 confidence level (ANOVA followed by Tukey HSD test).

Table S1. A list of the additional *Triticum* genotypes upon which gas exchange analysis was performed

Species name	Accession number/ cultivar	Ploidy level	Domestication status
Triticum baeoticum	TRI 19028	2n	Wild
Triticum urartu	TRI 17128	2n	Wild
Triticum araraticum	TRI 16599	4n	Wild
Triticum dicoccoides	TRI 18530	4n	Wild
Triticum durum	Aventadur	4n	Domesticated
Triticum durum	Surmesur	4n	Domesticated
Triticum aestivum	Cashel	6n	Domesticated
Triticum aestivum	Conqueror	6n	Domesticated
Triticum aestivum	Conversion	6n	Domesticated
Triticum aestivum	Gallant	6n	Domesticated
Triticum aestivum	Sacramento	6n	Domesticated
Triticum aestivum	Scout	6n	Domesticated
Triticum aestivum	Shamrock	6n	Domesticated
Triticum aestivum	Skyfall	6n	Domesticated
Triticum aestivum	Stigg	6n	Domesticated
